# Changes in group behaviour in response to a preferred environment reflect positive affect

**DOI:** 10.1038/s41598-023-37763-0

**Published:** 2023-06-29

**Authors:** Tanja K. Kleinhappel, Thomas W. Pike, Oliver H. P. Burman

**Affiliations:** School of Life Sciences, University of Lincoln, Lincoln, LN6 7DL UK

**Keywords:** Animal behaviour, Ichthyology

## Abstract

When observed in their preferred environments, animals display behavioural changes, such as an increase in resting or a reduction in agonism, suggestive of positive affect and improved welfare. However, most studies focus on the behaviour of individuals or, at most, pairs of animals; even though in group-living animals beneficial environmental changes may impact on how the group behaves as a whole. In this study, we investigated whether experiencing a preferred visual environment affected the shoaling behaviour of zebrafish (*Danio rerio*) groups. We first confirmed a group preference for an image of gravel placed underneath the base of a tank compared to a plain white image. Second, we observed replicated groups either with or without the preferred (gravel) image present to determine if a visually enriched and preferred environment could elicit changes in shoaling behaviour. We found a significant interaction between the observation time and test condition, with differences in shoaling behaviour reflective of increased relaxation emerging gradually over time in the gravel condition. The findings of this study reveal that experiencing a preferred environment can alter group behaviour, making such holistic changes valuable as potential indicators of positive welfare.

## Introduction

Animals have been shown to adapt their behaviour according to the environmental context they are presented with^1^, and these behavioural differences are evident at both the individual and the group level^2^. Such behavioural changes can be caused by social, ecological and environmental factors. For instance, the social environment, such as group composition (i.e. the presence or absence of conspecifics or heterospecifics) and group size, can alter both individual behaviour and interactions within groups (e.g.^3–6^), and recent social experience can influence personality expression^7^. Group dynamics can further be influenced by the ecological context the animals are observed in. Group cohesion and activity, for example, have been shown to differ depending on whether animals are foraging or encountering the threat of predation (e.g.^8^). Finally, enrichment of the environment, such as the inclusion of physical structures, increase in available space, provision of foraging opportunities and sensory stimulation can be implemented in order to promote positive effects on the behaviour and welfare of individuals and groups^9^.

In fish, for example, studies have shown that increasing structural complexity, such as the addition of substrates (e.g. gravel, sand), plants (artificial or live), or other features (e.g. shelters in form of pipes, caves)^10,11^ can decrease aggressive behaviour^12^, impact on anxiety and stress responses^13^ and aid the recovery from stressful situations^14^, see^15^ for a review. However, other environmental manipulations can also affect group dynamics in fish. For instance, increased space availability has been shown to decrease group density by increasing inter-individual distances^16^, while comparatively small increases in water turbidity can result in smaller group sizes and lower activity levels^17^ and weak water flow can increase aggression and decrease shoal cohesion^18^. Overall, these findings have important implications for management and welfare in captive animals, as they can give insight into the behavioural responses of animals to environmental/husbandry modifications implemented in order to improve animal welfare, as well as identifying potential behavioural indicators of stress and welfare.

Intriguingly, however, studies have shown that animals also respond to solely visual changes in their environment, without any concomitant change to physical structure. Several species of fish, for example, have been shown to prefer dark over light environments^19,20^, while zebrafish (*Danio rerio*) have individual preferences for blue and green environments over red and yellow ones^21^—although these results can be heterogeneous^22^. Preferences for achromatic horizontal, vertical and square patterns depend on the precise size of the pattern elements^23^. Possibly of more ecological relevance, zebrafish have also been shown to have a preference for images of gravel placed underneath the testing tank, spending a comparable amount of time in a compartment containing a gravel image compared to a compartment containing actual gravel^10^.

In most of the above studies only individual choice behaviour was assessed, and even when groups were tested (e.g.^10^) the focus was not typically on shoaling behaviour. However, in highly social species changes in shoaling behaviour can be seen as a flexible response to differing environmental conditions^8,17,18,24^. For instance, we previously showed that zebrafish shoal closer together when exposed to a novel (negatively stressful) environment, but tend to spread out as they become habituated^24^. In the current study, using zebrafish as a model, we aimed to test whether a visually enriched environment could elicit shoaling behaviour potentially indicative of positive affect and thus welfare, i.e. behaviour reflecting the presence of positive, rather than solely the absence of negative, experiences (e.g.^25^). We first looked to confirm that groups of fish preferred a gravel image compared to a plain white image placed under the base of a tank, as previously found^10^. Second, we observed replicated shoals in a test tank featuring the preferred (gravel) image and compared their shoaling behaviour to replicated shoals observed in a test tank featuring the non-preferred (plain white) image. We predicted that groups tested with the preferred image would show differences in shoaling behaviour indicative of a positive experience, compared to shoals tested with the non-preferred image.

## Methods

### Animals and housing

Adult wildtype zebrafish, obtained from a home aquarium supplier (Aquatics to your Door, UK), were housed in mixed-sex groups of around 35 fish in the aquatics facility at the University of Lincoln (UK). On arrival, fish were randomly allocated to replicate unenriched holding tanks measuring 52 × 44 × 31 cm and filled with 35 l of dechlorinated and UV-sterilised water. Water was maintained at a constant temperature of 24 ± 1 °C, which is consistent with temperatures experienced by zebrafish naturally in the wild^e.g.^^26^, and the photoperiod maintained on a 12:12 light:dark cycle (on: 6:00 h, off: 18:00 h) provided by ceiling-mounted fluorescent lights. Fish were fed daily to saturation with defrosted Chironomid larvae (bloodworms). They were kept in these conditions for at least 3 months before the start of the study, at which time their mean ± SD standard length was 37.6 ± 1.96 mm. This study followed the ARRIVE guidelines^27^ and all methods used adhered to the ASAB Guidelines for the Use of Animals in Research and gained local institutional ethical approval by the Research Ethics Committee of the University of Lincoln (UID CoSREC211).

### Preference test

In order to confirm that our population of fish preferred a tank with an image of gravel placed under its base compared to a white image, as has been shown previously for this species^10^, replicated groups of zebrafish (a total of 7 independent groups with 7 fish per group, as in^24^ were tested in a two-chamber glass choice tank 45 × 25 × 25 cm (L × W × H) filled with aerated dechlorinated water to a depth of 20 cm (22.5 L). The tank was divided into two equally sized partitions using an opaque PVC foam board, with a 5 × 5 cm opening at a height of 1.5 cm from the bottom of the tank allowing the fish to move between the two compartments. In one of these compartments, a laminated colour image of gravel was placed underneath the transparent base of the tank. This image was the same as that used previously^10^, but repeated so that it fitted under the whole area of the testing tank. The other compartment contained a laminated sheet of plain white paper. The order of the side (left or right compartment) in which the gravel image was presented was randomised (via a coin toss) to avoid a possible side bias of the fish. The test tank was visually separated from adjacent tanks using a white PVC board located approximately 10 cm away from the tank walls.

Before the start of the experimental session, seven fish were randomly selected from the same housing tank and assigned randomly to one of the two compartments, with four individuals being placed into one compartment and the remaining three in the other; which side received the four fish was randomly selected so that, on average, there was no initial bias towards any particular side. Shoals were then filmed for 60 min using a camera mounted above the experimental tank. Video recording started immediately before releasing the fish, and the experimenter left the room within 30 s. Each individual fish (and hence each group) was tested only once. To quantify preferences over time, the proportion of fish in the compartment containing the gravel image was determined from the recorded video at 30 s intervals.

### Behavioural test

To investigate possible behavioural differences in response to the preferred image, replicated groups of fish (30 independent groups in total, with 7 naïve fish per group) were placed in a 45 × 25 × 25 cm (L × W × H) experimental tank containing either the (preferred) gravel image (n = 14 groups, because one group had to be excluded due to a video failure) or the (unpreferred) plain white image (n = 15 groups). Experimental conditions were otherwise identical to those used during the preference tests.

After releasing the fish gently into the centre of the testing tank, shoals were filmed for the next 60 min using the overhead camera. Images were extracted from the video footage at 10 s intervals, and the two-dimensional position of each individual fish in a shoal was manually extracted using custom-written Matlab (MathWorks, Natick, MA) code^28^, and used to compute shoal density (a metric that we have previously shown is indicative of acute stress in zebrafish^24^) for each frame. Shoal density was defined as the number of individuals associating divided by the total number of possible associations within the shoal, where we considered an association as two fish being within two body lengths (twice the mean body length of all fish in the shoal) of each other^24^. This distance is within the range of inter-individual distances observed in free-ranging shoals^29^ and has previously been used to characterise social associations in fish^30–33^.

### Statistical analysis

All analyses were conducted in R version 4.0.4 (R Core Development Team). For the preference test, we tested whether fish preferred the compartment containing the gravel image compared to the compartment containing the plain white image as a function of time by fitting a non-linear mixed-effects model (using the nlmer function in the lme4 package^34^) in which the response variable (the relative proportion of fish in the gravel compartment) was related to time using an asymptotic function. Group identity was included as a random effect to control for repeated measures over time. To describe temporal changes in preference, we tested whether the intercept (representing initial preferences) and the asymptote (representing subsequent preferences) of the non-linear model differed significantly from chance (i.e., a preference of 0.5) using Wald tests^35^.

For the behavioural test, we tested whether shoal density differed when the floor of the test tank contained the (preferred) gravel image compared to the (unpreferred) plain white image by fitting a general linear mixed-effects model (using the lmer function in the lme4 package) with shoal density as the response variable, and the interaction between condition (white or gravel floor) and time as the predictor. The response variable was square root transformed in order to ensure normality of the model residuals, and the model included by-group random slopes. The significance of the interaction was tested by comparing the full model against a null model containing only the main effect terms using a likelihood ratio test^36^, and the normality and homoscedasticity of residuals confirmed visually^37^. Similar general linear mixed-effects models were subsequently used to explore the interaction in more detail, by testing for changes in shoal density over time for each condition separately.

## Results

### Preference test

**Figure 1:**
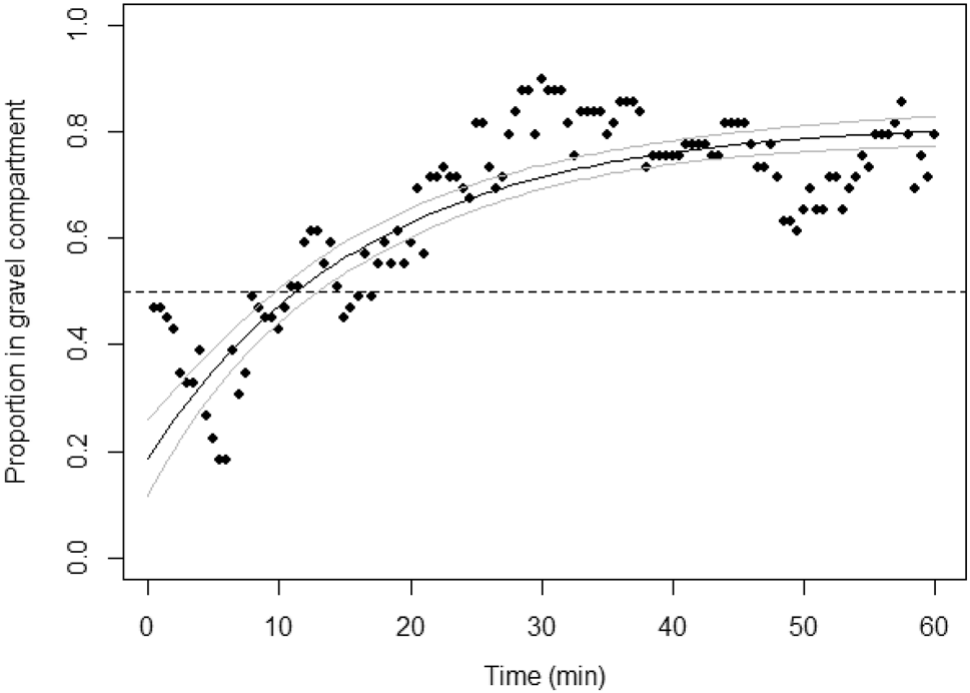
Proportion of fish in the gravel compartment as a function of time (n = 7 groups). For clarity, data points show the mean over all seven groups for a given time point. The solid line denotes the asymptotic fit from the non-linear mixed-effects model, the grey lines show the bootstrapped 95% confidence intervals, and the dashed line indicates chance preference levels.

When presented with a simultaneous choice between two otherwise identical compartments, one with a plain white image underneath and the other with an image of gravel underneath, fish initially preferred the white image (i.e., the intercept in the non-linear model was significantly lower than chance: estimate ± SE, 0.23 ± 0.04; z = –6.64, p < 0.001) although over time this changed to a significant and sustained preference for the compartment with the gravel image (i.e., the asymptote in the non-linear model was significantly greater than chance: estimate ± SE, 0.80 ± 0.02; z = 13.85, p < 0.001) (Fig. 1).

### Behavioural test

**Figure 2:**
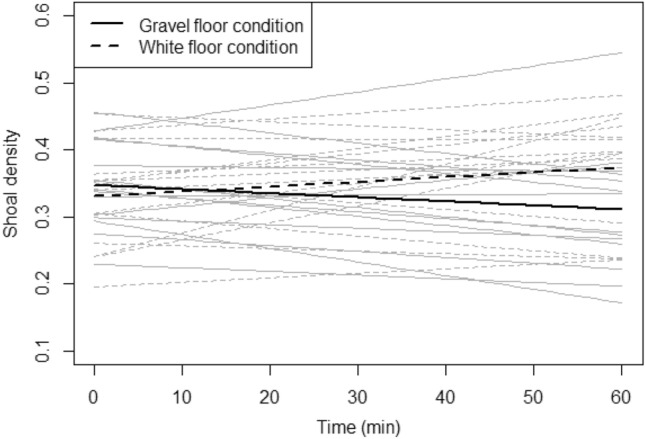
Shoal density as a function of time in the gravel floor condition (solid lines, n = 14 groups) and the white floor condition (dashed lines, n = 15 groups). The thick black lines denote the fit from the linear mixed-effects models, and the grey lines the fits from models for individual groups.

When comparing the shoaling behaviour of fish between the two separate conditions (a gravel or a plain white image placed under the tank), shoal density was significantly predicted by the interaction between condition and time (χ^2^(1) = 8.17, p = 0.004; Fig. 2). Specifically, while the intercepts were not significantly different to one another (i.e., there was no initial difference between conditions: z = 0.83, p = 0.404), there was a significant increase in shoal density over time in the white image condition (χ^2^(1) = 4.07, p = 0.044) and a significant decrease over time in the gravel image condition (χ^2^(1) = 4.18, p = 0.041).

## Discussion

This study investigated whether the presence or absence of a preferred environment could influence the group behaviour of animals. First, the results confirmed that the zebrafish groups had a preference for the presence of an image of gravel, compared to a plain white image. Second, when groups were tested in tanks containing either the preferred gravel image or the unpreferred white image, changes in their shoaling density over time were observed, reflective of increased relaxation in the gravel condition and increased anxiety in the white floor condition.

The preference for the gravel image is consistent with the findings of Schroeder et al.^10^, who found that zebrafish exhibit similar levels of preference for an image of gravel placed under their tank compared to an actual gravel substrate. Interestingly, in our preference test, fish initially appeared to avoid the compartment with the gravel image, which can be noted by the comparatively low average proportion of fish in the gravel compartment during the first 10 min (Fig. 1). We suggest that this initial avoidance is likely due to the novelty of the gravel image, which they had never previously encountered. Animals in preference tests typically require pre-exposure too, or significant experience of, a particular stimulus/environment before a preference for that stimulus/environment can be revealed^38^. Nonetheless, after this early avoidance, the fish subsequently spent the majority of their time in the compartment containing the gravel image, indicating that, once the image had become familiar, it was soon consistently preferred. Results in other species of fish have shown that they prefer a more complex substrate (e.g. different coloured pebbles or shell pieces) over a simple one containing, for instance, only fine sand (red snapper (*Lutjanus campechanus*)^39^; three-spined stickleback (*Gasterosteus aculeatus*)^40^). This suggests that a visually more heterogeneous and complex substrate might be advantageous for fish, possibly as a more effective background for avoiding predation (e.g. least killifish (*Heterandria formosa*)^41^; rock gobies (*Gobius paganellus*)^42^). This could either be because it resembles a natural substrate (zebrafish^43^) or because it makes the tank appear darker or deeper (zebrafish^44^), which is generally preferred as it is perceived as a safer environment (three-spined stickleback^45^). However, although there may be general principles that are translatable across species, it is also important to consider that there may be species-specific and/or individual/group preferences for certain features of enrichment. For example, rock gobies preferred the background that they were best at colour-matching morphologically^42^.

Following the preference test, we observed that when groups of fish were housed in an environment with only the preferred gravel image under the base of the tank, significant changes in their shoaling behaviour were observed over time. There was no initial difference in group behaviour between the conditions (gravel or white image), most likely because, as in the preference test, there was an initial (c. 10 min) behavioural stress response to the novel (gravel) environment—not previously experienced in their holding tanks. Once the fish had become familiar with the environment, however, they showed a change in group behaviour that appeared to reflect increased relaxation, similar to that observed in our previous study^24^ in which group behaviour changed as groups became habituated to an initially novel environment. However, in the current study the fish exposed to the preferred gravel image subsequently showed a significantly reduced shoal density compared to those in the white environment (which was also novel, and to which they would be expected to habituate). This suggests that they were not simply becoming less anxious with increased habituation, but that they showed a ‘positive’ behavioural response to experiencing the presence of the (preferred) gravel floor.

Such changes are comparable to studies showing that the preferred housing environments of animals (e.g. those containing plants and shelter) can promote resilience, including recovery from stressful situations^14^ and/or a reduced behavioural response to (negative) stress^46^, and mirrors research revealing how environmental enrichment provision induces behavioural expression of positive welfare and not just the removal/reduction of behavioural indicators of negative stress (e.g. laboratory rats^47^; broiler chickens^48^). However, there are reports of contrasting findings to ours in group response to stress. For example, Suriyampola et al.^18^ observed less cohesive groups when zebrafish were exposed to a weak water flow, and Powell et al.^49^ recorded a decrease in social cohesion immediately after exposure to tank cleaning. This suggests that group responses may vary according to context, such as the type, mode of delivery and timing of any given stressor, as well as showing sensitivity to valence; reflecting the variation in stress response that is also seen in individuals^50^ and highlighting the importance of including additional indicators alongside measures of group cohesion in order to aid overall interpretation. For example, in the apparent absence of stressors, zebrafish were found to show instances of ‘heightened-shoaling’ that may reflect a positive, rather than negative, emotional response given concurrent reductions in agonism and increased behavioural synchrony^51^. A group’s response to stress may also be influenced by their sex ratio or social history, with recently formed sub-groups of familiar individuals (as used here) behaving differently to more long-established stable social groups (e.g.^18,49^).

Overall, our study highlights the impact that simple visual manipulations of environmental factors can have on group behaviour and, thus, confirms the importance of enrichment provision for promoting good welfare and better quality animal models (e.g.^52,53^). Furthermore, it emphasises the importance of taking more holistic group responses into account when studying behaviour and welfare. Social interactions are central to group-living animals and, as such, should be considered when studying animal welfare as they can add valuable information in various different contexts, including the positive and negative impacts of an animal’s environment. For instance, taking the results of the current study and Kleinhappel et al.^24^ together, shoaling behaviour appears to be a valuable measure of stress that appears to reflect affective valence (i.e. positive/negative)—a key component of welfare assessment^54^—with negative affect leading to a higher, and positive affect to a lower, shoal density. Arousal effects on group behavioural changes should therefore be a future area of focus in order to determine whether group (as opposed to individual or dyadic) responses to stress can allow for more nuanced sensitivity.

## Data Availability

All the data presented in this paper will be made available in an open access repository immediately on acceptance.
